# Transcriptome profiling of whitefly guts in response to *Tomato yellow leaf curl virus* infection

**DOI:** 10.1186/s12985-018-0926-6

**Published:** 2018-01-16

**Authors:** Liang Geng, Li-Xin Qian, Ruo-Xuan Shao, Yin-Quan Liu, Shu-Sheng Liu, Xiao-Wei Wang

**Affiliations:** Ministry of Agriculture Key Lab of Molecular Biology of Crop Pathogens and Insects, Institute of Insect Sciences, Zhejiang University, Hangzhou, 310058 China

**Keywords:** Transcriptome, Whitefly, TYLCV, Gut, Gene expression

## Abstract

**Background:**

Plant viruses in agricultural crops are of great concern worldwide, and over 75% of them are transmitted from infected to healthy plants by insect vectors. *Tomato yellow leaf curl virus* (TYLCV) is a begomovirus, which is the largest and most economically important group of plant viruses, transmitted by the whitefly *Bemisia tabaci*. The circulation of TYLCV in the insect involves complex insect-virus interactions, whereas the molecular mechanisms of these interactions remain ambiguous. The insect gut as a barrier for viral entry and dissemination is thought to regulate the vector specificity. However, due to its tiny size, information for the responses of whitefly gut to virus infection is limited.

**Methods:**

We investigated the transcriptional response of the gut of *B. tabaci* Middle East-Asia Minor 1 species to TYLCV infection using Illumina sequencing.

**Results:**

A total of 5207 differentially expressed genes (DEGs) between viruliferous and non-viruliferous whitefly guts were identified. Enrichment analyses showed that cargo receptor and ATP-binding cassette (ABC) transporters were enriched in DEGs, and might help the virus to cross gut barrier. TYLCV could perturb cell cycle and DNA repair as a possible result of its replication in the whitefly. Our data also demonstrated that TYLCV can activate whitefly defense responses, such as antimicrobial peptides. Meanwhile, a number of genes involved in intracellular signaling were activated by TYLCV infection.

**Conclusions:**

Our results reveal the complex insect-virus relationship in whitefly gut and provide substantial molecular information for the role of insect midguts in virus transmission.

**Electronic supplementary material:**

The online version of this article (10.1186/s12985-018-0926-6) contains supplementary material, which is available to authorized users.

## Background

Plant viral diseases have received great attention worldwide because of their tremendous economic impact [1]. The majority of plant viruses are transmitted by insects of hemipteran families, such as aphids, whiteflies, leafhoppers, planthoppers, and thrips [2]. As a consequence, vector control is currently the only practical and effective strategy for disease prevention. Over the past few decades, a number of research have investigated the interactions between plant viruses and insect vectors because of its importance in viral epidemiology and disease management [2–4]. A detailed understanding of the genetic and molecular basis of insect-virus interaction will lead the discovery of novel and specific molecular targets for whitefly and whitefly-transmitted virus control.

*Tomato yellow leaf curl virus* (TYLCV) (Geminiviridae; *Begomovirus*) causes one of the most devastating emerging diseases of tomato worldwide [5]. TYLCV is transmitted by the whitefly *Bemisia tabaci* in a circulative persistent manner [2, 6]. *B. tabaci* is a cryptic species complex composed of at least 36 species [7]. In this species complex, the Middle East-Asia Minor 1 (Herein called MEAM1) species is highly invasive and a superior, co-adapted vector for begomoviruses. The epidemics of begomoviruses are usually associated with outbreaks of MEAM1 and the relationships between begomoviruses and whiteflies are complex [8]. When the MEAM1 viruliferous whiteflies were transferred onto non-host plants of the virus, the longevity and fecundity of the viruliferous whiteflies decreased [9]. This indicates that begomoviruses, in some cases, are insect pathogens. The transcriptional response of MEAM1 whiteflies to *Tomato yellow leaf curl China virus* (TYLCCNV) demonstrated that TYLCCNV can activate whiteflies’ immune response [10]. Further results showed that whiteflies use a variety of defense mechanisms to combat virus infection, such as autophagy and antimicrobial peptides (AMPs) [11, 12].

Circulative plant viruses move through the insect vector, from the gut lumen into the haemolymph or other tissues and finally into the salivary glands from where viruses are disseminated to new host plants during insect feeding [13]. In this process, midgut and salivary glands are the two major barriers that viruses have to overcome before successfully transmitted [2]. In fact, the gut barrier is the principal determinant for the ability of an insect species to transmit a virus. For instance, the greenhouse whitefly *Trialeurodes vaporariorum* is a non-vector of TYLCV, because the viruses can’t cross midgut into haemolymph [14]. Persistent viruses, whether propagative or nonpropagative, can be transmitted to plants after injection into the insect hemolymph [15]. In many cases, injected viruses are transmitted at higher rates than orally acquired viruses [16, 17]. Microscopic studies have shown that TYLCV virions is extensively localized in the filter chamber and cross the epithelial cells in the midgut [6, 18]. Compared to the whole body of whiteflies, TYLCV has a longer retention and higher quantity in the midgut [19]. Nevertheless, TYLCV infection can activate the autophagy pathway in whitefly midguts, which inhibits the efficiency of virus transmission [11]. These studies show that midguts are major reservoir where virions accumulate during acquisition and are critical in insect-virus interaction. However, due to the small size of whitefly midgut, the transcriptional responses of whitefly gut to virus infection remains unknown.

With the development of sequencing technique, next generation sequencing have provided us a valuable tool for exploring transcriptional changes using less than 1 μg RNA samples. In this study, we extracted 700 ng RNA from approximately 1000 whitefly guts for RNA-Seq to examine changes in gene transcription between viruliferous and nonviruliferous whiteflies. Transcriptome analysis revealed that a number of genes involved in the material transport, cell cycle, DNA repair, defense responses, signaling molecules and pathways were regulated in the viruliferous whitefly guts. These data provide a valuable source of molecular information for the research of guts in TYLCV-whitefly interactions. To our knowledge, this is the first report to study the direct effect of a plant virus on global gene expression profile of vector’s gut using a high-throughput sequencing method.

## Results

### Sequence assembly and functional annotation

**Fig. 1:**
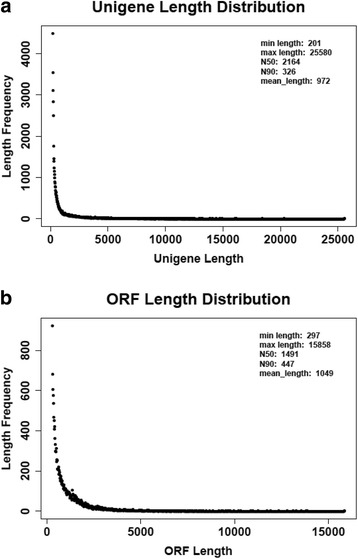
The length distribution of unigenes and ORFs. The x axis shows the lengths calculated in our library and the y axis shows the frequency. **a** The length distribution of unigenes. **b** The length distribution of ORFs

**Fig. 2:**
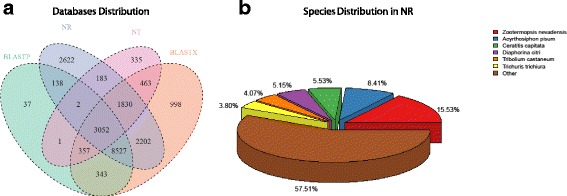
Distribution of databases and species. **a** Venn diagram showing databases distribution of unigenes (*E*-value< 10^−5^). NT: 6623 unigenes; NR: 18,556 unigenes; BLASTP: 12,457 unigenes and BLASTX: 17,772 unigenes. **b** Species distribution of unigenes in NR databases

Two cDNA libraries of viruliferous and nonviruliferous guts were sequenced and generated 51,471,400 and 51,554,406 raw reads, respectively. After filtering for high quality sequences, 44,362,142 and 45,610,802 clean reads were obtained for each sample (Table 1). Subsequently, the clean reads from the two samples were combined to do de novo assembly, resulting in 69,836 contigs with a mean length of 1121 bp and an N50 of 2407 bp. The lengths of contigs ranged from 200 bp to over 25,000 bp. After clustering, these contigs generated 55,124 unigenes (Fig. 1a). From these unigenes, a total of 18,574 predicted ORFs was obtained and the lengths ranged from 297 bp to 15,858 bp, with a mean length of 1049 and an N50 of 1491 (Fig. 1b). To annotate the unigenes, we searched reference sequences against NT, NR, PFAM and Uniprot databases, using Trinotate and Blast with a cut-off *E*-value of 10^− 5^. 21,259 (38.57%) unigenes were annotated at least in one database, including 6623 in NT, 18,556 in NR, 12,457 in BLASTP and 17,772 in BLASTX against Uniprot (Fig. 2a). On the basis of NR annotation, 15.53% (*n* = 2882) of unigenes matched to *Zootermopsis nevadensis*, followed by *Acyrthosiphon pisum*: 8.40% (*n* = 1560); *Ceratitis capitata*: 5.53% (*n* = 1026); *Diaphorina citri*: 5.15% (*n* = 956); *Tribolium castaneum*: 4.07% (*n* = 755) (Fig. 2b).

**Table 1:** Statistics of DEG sequencing

Category	viruliferous guts	nonviruliferous guts
Raw Reads Number	51,471,816	51,554,406
Clean Reads Number	44,362,142	45,610,802
Clean Reads Rate (%)	86.19	88.47
Clean Q30 Bases Rate (%)	96.06	96.43

Two cDNA libraries were sequenced using Illumina Hiseq 4000 platform. The raw data were transformed into the clean data using Perl scripts. The Q30 bases is the bases quality more than 30

### Global patterns of gene expression in response to TYLCV infection

**Fig. 3:**
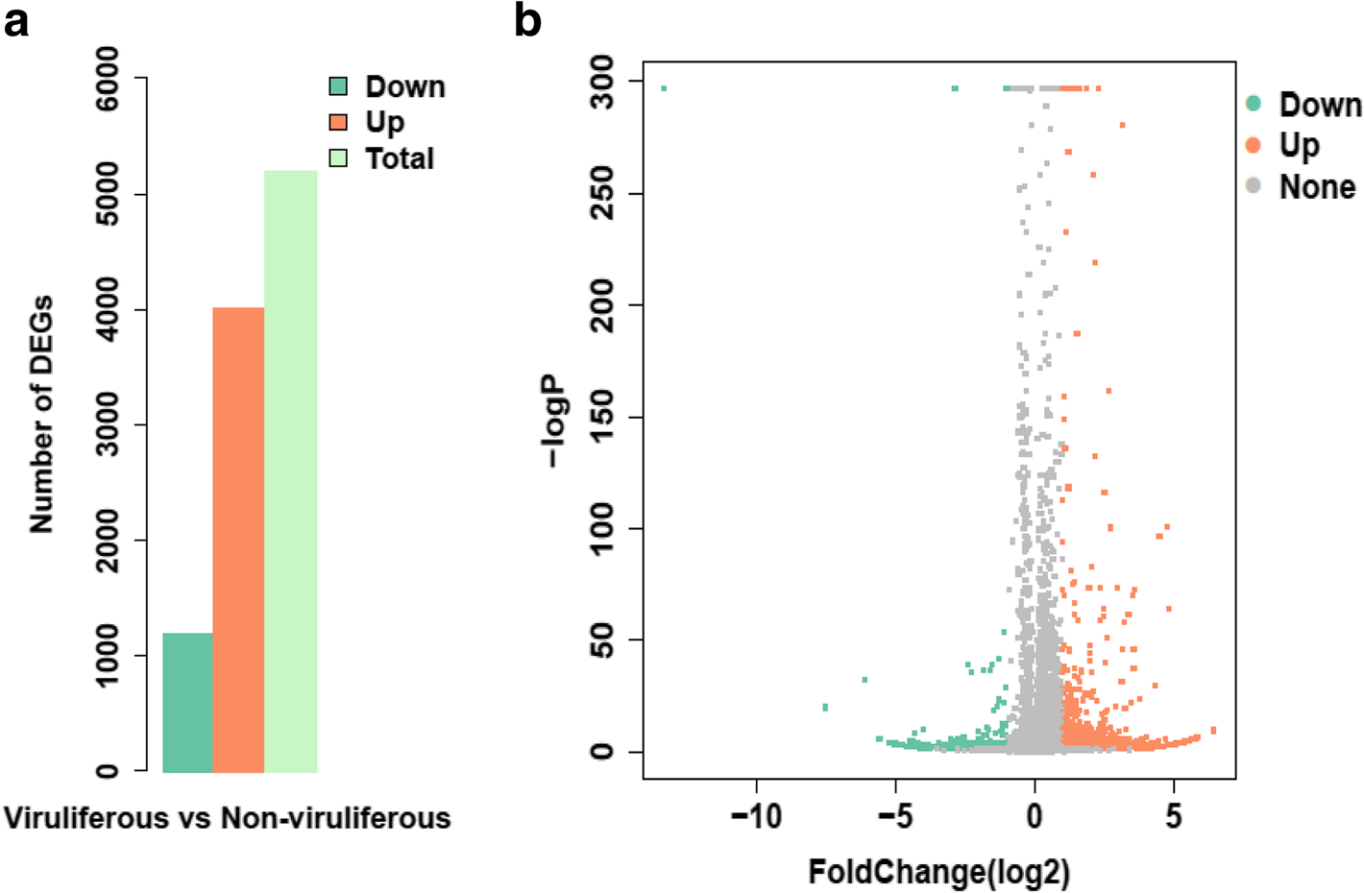
Analysis of differentially expressed genes between two libraries. **a** Summary of the numbers of differentially expressed genes in the TYLCV viruliferous whitefly guts. Genes with q ≤ 0.05 (adjusted *p*-value) and log_2_ ratio ≥ 1 were considered differentially expressed. **b** Correlation analysis of fold change and *p*-value

Subsequently, the differentially expressed genes (DEGs) between viruliferous and nonviruliferous guts were identified using DEGseq [20]. A total of 5207 differentially expressed genes were identified, with 4014 genes upregulated and 1193 genes downregulated in viruliferous whitefly guts (Fig. 3a). The detected fold changes (log_2_ ratio) of gene expression ranged from − 13.31 to 6.45, and more than 90% of the genes (4696) were up- or downregulated between 1.0- and 4.0-fold (Fig. 3b).

### Assignment of DEGs to Gene Ontology (GO) terms and Kyoto Encyclopedia of Genes and Genomes (KEGG) pathways

**Fig. 4:**
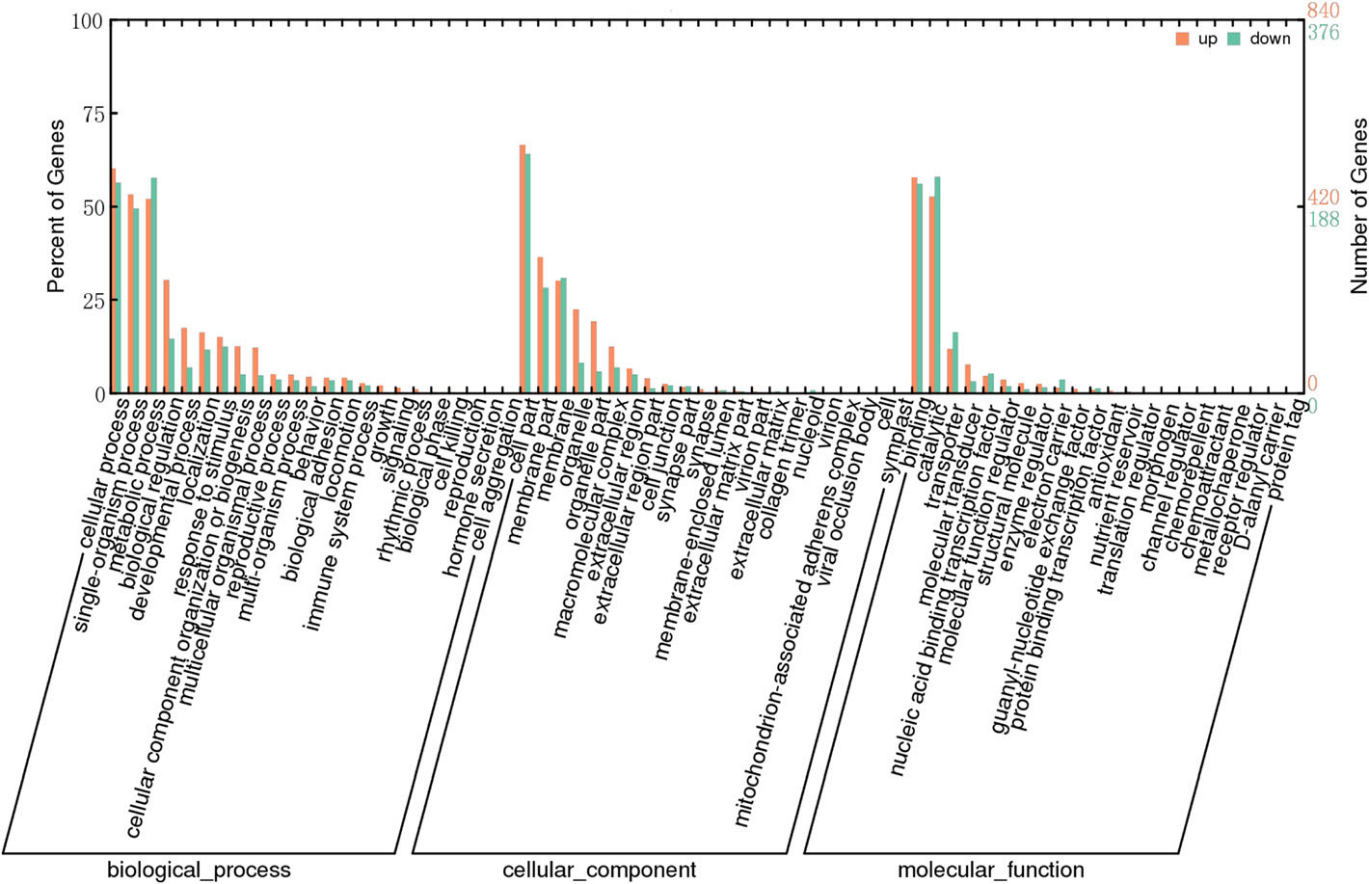
Histogram presentations of GO classificarion of differentially expressed genes.The functions of genes indentified cover three main categories: biological process, cellular component and molecular function. GO analysis showed that the distributions of gene functions for the differentially expressed genes

To further reveal their functions, GO assignments were used to classify the DEGs. A total of 1216 DEGs (840 upregulated and 376 downregulated) were categorized into 54 secondary GO categories under the ‘biological process’, ‘cellular component’ and ‘molecular functions’ divisions. While ‘binding’ and ‘catalytic’ were among the most represented ‘molecular functions’ categories, the ‘cellular component’ most represented were ‘cell part’ and ‘membrane part’. Interestingly, in ‘biological process’ category, viral infections dramatically changed the expression of genes in ‘response to stimulus’, ‘reproductive process’ and ‘immune system process’ (Fig. 4). The GO enrichment analysis showed that ‘Cargo receptor activity’ was significantly enriched with DEGs in ‘molecular functions’ (11 out of 30 genes). Exactly 6 genes were upregulated while a gene was downregulated in ‘scavenger receptor activity’ under the ‘cargo receptor activity’ (Table 2).

**Table 2:** DEGs involved in the scavenger receptor activity

Category or gene ID	Homologous function^*a*^	Species	FC^*b*^
c18796_g2	Somatomedin-B and thrombospondin protein	*Acyrthosiphon pisum*	1.4
c20133_g1	Aatrial natriuretic peptide-converting enzyme	*Fopius arisanus*	1.6
c22574_g4	Enteropeptidase	*Zootermopsis nevadensis*	1.9
c26558_g2	Scavenger receptor class B member 1	*Acyrthosiphon pisum*	2.8
c3386_g1	Lysyl oxidase homolog 3	*Megachile rotundata*	4.2
c36420_g2	Lysyl oxidase homolog 2	*Acyrthosiphon pisum*	1.3

Displayed are the fold changes of DEGs involved in the scavenger receptor activity of viruliferous guts in comparison to nonviruliferous guts. log2 ratio ≥ 1 and q ≤ 0.05 are used as the threshold

^*a*^The function of the homologous gene

^*b*^*FC*, fold change (log_2_ ratio) of gene expression

Among the 5207 DEGs, 414 genes were mapped to 266 pathways in KEGG database. While ‘metabolic pathways’ (169), ‘biosynthesis of secondary metabolites’ (50) and ‘microbial metabolism in diverse environments’ (37) contained most of the DEGs. Enrichment analysis demonstrated that the DEGs were only significantly enriched in ‘ATP-binding cassette (ABC) transporters’, with 16 genes upregulated and 17 genes downregulated in viruliferous whitefly guts.

Based on GO and KEGG analysis, a total of 31 genes (24 upregulated and 7 downregulated) involved in cell cycle were found in our DEGs (Table 3). For DNA repair, 2 genes were upregulated and 6 genes downregulated in viruliferous whitefly guts (Table 4). Additionally, 4 genes of cell cycle checkpoints were found in DEGs and all of them were upregulated (Table 4). We also found that 10 upregulated genes were related to ubiquitin (Table 4).

**Table 3:** DEGs involved in cell cycle

Category or gene ID	Homologous function^*a*^	Species	FC^*b*^
Cell cycle			
c30965_g3	Cell division cycle protein 16 homolog	*Aedes aegypti*	1.1
c43556_g1	G1/S-specific cyclin-D2	*Zootermopsis nevadensis*	2.2
c9437_g2	Chromosomal replication initiator protein	*Bombus impatiens*	3.2
c22603_g4	DNA translocase FtsK	*Plutella xylostella*	−1.7
c20875_g1	Transcription factor Sox-2	*Diaphorina citri*	1.0
c27290_g1	Serine/threonine-protein kinase greatwall	*Plutella xylostella*	1.3
c36551_g2	TATA element modulatory factor	*Pediculus humanus*	1.8
c39176_g1	FtsL and Transpeptidase and PBP domain protein	*Trichuris trichiura*	3.9
c31762_g2	Centrosomal protein of 152 kDa-like isoform X2	*Camponotus floridanus*	1.1
c42462_g1	Cell division activator CedA	*Shigella*	−1.8
c22785_g1	Regulator of sigma E protease	*Ceratosolen solmsi marchali*	−3.1
c9110_g1	Lon protease homolog 2, peroxisomal-like	*Plutella xylostella*	−2.5
c19824_g2	Bifunctional protein BirA	*Ceratitis capitata*	−1.2
c23042_g1	Mmultiple endocrine neoplasia type 1	*Lymnaea stagnalis*	2.0
c27223_g1	Protein patched, partial	*Zootermopsis nevadensis*	1.1
c46096_g1	Cell division protein ZipA homolog	*Shigella*	−1.8
c19557_g2	Muramoylalanine--D-glutamate ligase	*Ceratitis capitata*	3.1
c9059_g2	Polycomb protein Asx isoform X1	*Acyrthosiphon pisum*	1.8
c27863_g2	Protein vestigial-like	*Diaphorina citri*	1.2
c23930_g2	Maternal embryonic leucine zipper kinase-like	*Diaphorina citri*	2.5
c28988_g1	Neurogenic locus notch homolog protein 2	*Acyrthosiphon pisum*	1.0
c33849_g2	Protein abnormal spindle	*Diaphorina citri*	2.3
c15572_g1	Methylcytosine dioxygenase TET2	*Fopius arisanus*	2.3
c36390_g2	Protein lap4-like	*Diaphorina citri*	1.2
c30627_g2	Transposase	*Lasius niger*	2.6
c12143_g2	MMR HSR1 domain containing protein	*Trichuris trichiura*	3.6
c23930_g1	Maternal embryonic leucine zipper kinase	*Zootermopsis nevadensis*	3.0
c19933_g4	MAP kinase-activating death domain protein	*Fopius arisanus*	2.5
c19501_g2	GAS2-like protein 1	*Acyrthosiphon pisum*	1.8
c7159_g1	Homeobox protein cut	*Fopius arisanus*	−3.7
c34320_g11	Ubiquitin-like-specific protease 1	*Schizosaccharomyces*	1.5

Displayed are the fold changes of DEGs involved in cell cycle of viruliferous guts in comparison to nonviruliferous guts. log2 ratio ≥ 1 and q ≤ 0.05 are used as the threshold

^*a*^The function of the homologous gene

^*b*^FC, fold change (log_2_ ratio) of gene expression

**Table 4:** DEGs involved in DNA repair

Category or gene ID	Homologous function^*a*^	Species	FC^*b*^
DNA repair			
c45190_g1	DNA ligase aden and DNA ligase OB domain	*Trichuris trichiura*	−1.5
c39710_g1	DNA polymerase I	*Trichuris trichiura*	−1.8
c23489_g3	DNA polymerase III subunit alpha	*Capitella teleta*	−1.7
c30501_g2	DNA polymerase III subunit epsilon	*Capitella teleta*	2.0
c20205_g4	ATP-dependent DNA helicase srs2-like	*Plutella xylostella*	3.1
c20205_g1	ATP-dependent DNA helicase srs2-like	*Plutella xylostella*	2.6
c4255_g1	MutS protein-like protein 4, partial	*Zootermopsis nevadensis*	−3.7
c21553_g1	DNA glycosylase	*Lasius niger*	−2.4
c37356_g1	Uracil-DNA glycosylase	*Capitella teleta*	−2.1
Cell cycle checkpoints			
c32780_g1	RAD50-interacting protein 1	*Zootermopsis nevadensis*	1.0
c34907_g2	Cell cycle checkpoint protein RAD1 isoform X2	*Cerapachys biroi*	1.0
c23966_g1	Transformation/transcription domain protein	*Zootermopsis nevadensis*	2.5
c15302_g2	Transformation/transcription domain protein	*Acyrthosiphon pisum*	3.6
E3 ubiquitin ligase			
c37868_g1	G2/M phase-specific E3 ubiquitin-protein ligase	*Danio rerio*	2.4
c28057_g2	G2/M phase-specific E3 ubiquitin-protein ligase	*Saccoglossus kowalevskii*	1.6
c14143_g1	G2/M phase-specific E3 ubiquitin-protein ligase	*Danio rerio*	1.7
c37987_g1	G2/M phase-specific E3 ubiquitin-protein ligase	*Gallus*	2.9
c50210_g1	G2/M phase-specific E3 ubiquitin-protein ligase	*Crassostrea gigas*	1.6
c35060_g2	E3 ubiquitin-protein ligase HUWE1	*Daphnia pulex*	1.2
c13801_g1	Hect E3 ubiquitin ligase	*Pediculus humanus*	1.1
c27270_g1	E3 ubiquitin-protein ligase BRE1-like	*Diaphorina citri*	1.1
c29883_g1	NEDD4-like E3 ubiquitin-protein ligase WWP1	*Zootermopsis nevadensis*	1.0
c31381_g2	Apoptosis-resistant E3 ubiquitin protein ligase 1	*Athalia rosae*	1.0

Displayed are the fold changes of DEGs involved in DNA repair of viruliferous guts in comparison to nonviruliferous guts. log2 ratio ≥ 1 and q ≤ 0.05 are used as the threshold

^*a*^The function of the homologous gene

^*b*^FC, fold change (log_2_ ratio) of gene expression

Interestingly, among the DEGs in viruliferous whitefly guts, 14 genes related to cellular and humoral immune response were upregulated (Table 5). Six genes encoding AMPs were significantly upregulated including 1 defensin and 4 knottin. For cellular responses, a total of 8 upregulated genes were found, 6 of them involved in the phagocytosis and 2 in encapsulation. In addition, 9 genes (6 upregulated and 3 downregulated) were altered in the lysosome pathway (Additional file 1: Table S1). For regulators of defense responses, 1 gene encoding secreted molecule and 9 genes encoding putative transmembrane receptors (surface receptors) were found in our DEGs (Table 5). Meanwhile, the intracellular signaling molecules or pathways, downstream of these receptors, were also activated. In viruliferous whitefly gut cells, a number of genes involved in the mitogen-activated protein kinase (MAPK), Notch, transforming growth factor beta (TGF-β), PI3K-Akt, Jak-STAT, protein kinase C (PKC), and Ras signaling molecules or pathways were upregulated (Table 6).

**Table 5:** Upregulation of genes involved in defence responses and regulators in viruliferous whitefly guts

Category or gene ID	Homologous function^*a*^	Species	FC^*b*^
Antimicrobial peptides			
c24521_g1	Antimicrobial knottin protein Btk-1	*Bemisia tabaci*	2.8
c29023_g1	Antimicrobial knottin protein Btk-2	*Bemisia tabaci*	4.8
c25386_g1	Antimicrobial knottin protein Btk-3	*Bemisia tabaci*	2.2
c27948_g1	Antimicrobial knottin protein Btk-4	*Bemisia tabaci*	2.6
c29424_g2	Defensin	*Bemisia tabaci*	6.5
c27979_g1	Putative defense protein 3	*Antheraea mylitta*	1.1
Phagocytosis			
c27585_g1	Pre-mRNA-splicing factor protein	*Zootermopsis nevadensis*	1.3
c31536_g2	Peroxidase	*Acyrthosiphon pisum*	1.0
c51435_g1	V-type proton ATPase subunit E 1	*Myotis brandtii*	3.1
c23664_g1	Tubulin beta-4 chain	*Xenopus*	1.0
c37031_g1	V-type proton ATPase subunit E-like, partial	*Diaphorina citri*	2.4
c2500_g1	MHC class I related secreted antigen, partial	*Mus musculus*	3.4
Encapsulation			
c22725_g1	Neuroglian precursor, putative	*Pediculus humanus*	2.1
c43096_g1	Dynein heavy chain, cytoplasmic-like	*Bombyx mori*	1.1
Humoral receptors			
c20023_g1	Beta-1,3-glucan recognition protein 4a	*Anasa tristis*	1.2
EGF-like repeats			
c27788_g2	Laminin subunit alpha-1	*Acyrthosiphon pisum*	−2.9
c43077_g1	Low-density lipoprotein receptor-related protein 1	*Zootermopsis nevadensis*	1.7
c28427_g2	Multiple epidermal growth factor-like domains 8	*Zootermopsis nevadensis*	1.1
CD36 family			
c26558_g1	Scavenger receptor class B member 1	*Zootermopsis nevadensis*	2.9
c24130_g1	Scavenger receptor class B member, putative	*Pediculus humanus*	1.2
c44985_g1	Scavenger receptor class B member 1-like	*Acyrthosiphon pisum*	3.5
c12821_g1	Scavenger receptor class B member, putative	*Pediculus humanus*	1.3
Toll receptors			
c22610_g3	Protein spaetzle	*Zootermopsis nevadensis*	5.5
c24421_g1	Protein toll precursor, putative	*Pediculus humanus*	1.3

Displayed are the fold changes of upregulation of genes involved in defence responses and regulators of viruliferous guts in comparison to nonviruliferous guts. log2 ratio ≥ 1 and q ≤ 0.05 are used as the threshold

^*a*^The function of the homologous gene

^*b*^FC, fold change (log_2_ ratio) of gene expression

**Table 6:** Intracellular signaling molecules and pathways involved in defence response

Category or gene ID	Homologous function^*a*^	Species	FC^*b*^
Ras signaling pathway			
c22269_g2	Glutamate [NMDA] receptor subunit 1	*Zootermopsis nevadensis*	1.1
c28829_g5	Neurofibromin	*Tribolium castaneum*	1.5
c25358_g2	Phosphodiesterase	*Stegodyphus mimosarum*	1.9
c43077_g1	Low-density lipoprotein receptor-related protein 1	*Zootermopsis nevadensis*	1.7
c25635_g3	Ephrin-B1	*Acyrthosiphon pisum*	2.1
c5499_g1	Collagen alpha-1(IV) chain	*Macaca fascicularis*	1.9
PI3K-Akt signaling pathway			
c1715_g1	Laminin subunit alpha-like protein, partial	*Lasius niger*	3.2
c36441_g1	Laminin subunit alpha, partial	*Zootermopsis nevadensis*	1.8
c33202_g1	Insulin receptor	*Zootermopsis nevadensis*	1.6
c23289_g2	Immunoglobulin superfamily member 10-like	*Diaphorina citri*	−2.1
Jak-STAT signaling pathway			
c35885_g1	Cytokine receptor	*Orussus abietinus*	1.5
MAPK signaling pathway			
c35895_g15	Transcription factor AP-1	*Zootermopsis nevadensis*	1.0
c28281_g1	Mitogen-activated protein kinase kinase 7	*Acyrthosiphon pisum*	1.5
c21772_g2	ETS domain-containing protein Elk-1	*Sarcophilus harrisii*	2.5
Protein kinase C			
c32967_g6	Protein kinase C-like	*Tribolium castaneum*	1.1
c21894_g1	Diacylglycerol kinase theta isoform X2	*Orussus abietinus*	2.2
Notch signaling pathway			
c34262_g4	Protein groucho isoform X5	*Tribolium castaneum*	1.2
c13638_g2	Fringe glycosyltransferase, putative	*Pediculus humanus*	1.6
c41024_g1	Delta-like protein C	*Pediculus humanus*	3.4
TGF-beta signaling pathway			
c24093_g1	TGF-beta receptor type-1	*Riptortus pedestris*	1.4
c42587_g1	Inhibin beta chain-like	*Diaphorina citri*	3.5
c43252_g1	Chordin	*Panthera tigris altaica*	3.9
c23496_g1	Dorsal-ventral patterning protein Sog-like, partial	*Monomorium pharaonis*	−1.8

Displayed are the fold changes of Intracellular signaling molecules and pathways involved in defence response of viruliferous guts in comparison to nonviruliferous guts. log2 ratio ≥ 1 and q ≤ 0.05 are used as the threshold

^*a*^The function of the homologous gene

^*b*^FC, fold change (log_2_ ratio) of gene expression

### qRT-PCR validation and expression profiling of DEGs

**Fig. 5:**
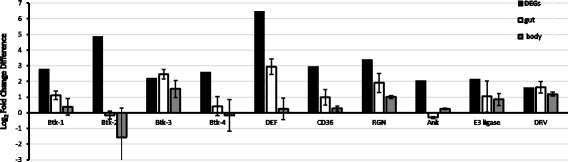
Result of RT-PCR. 10 selected genes were measured the expression in both guts and whole body using comparative C_T_ (ΔΔC_T_) qPCR with β-actin as the internal control gene. 10 genes were 5 antimicrobial peptides (Btk-1, Btk-2, Btk-3, Btk-4, DEF), 1 scavenger receptor class B (CD36), 1 regucalcin (RGN), 1 ankyrin repeat protein (Ank), 1 E3 ubiquitin-protein ligase, and 1 unnamed protein (categorized into go biological process, defense response to virus, DRV)

To validate our data, the expression levels of 10 DEGs were verified using qRT-PCR. We analyzed the expression of 5 antimicrobial peptides (Btk-1, Btk-2, Btk-3, Btk-4, DEF), 1 scavenger receptor class B (CD36), 1 regucalcin (RGN), 1 ankyrin repeat protein (Ank), 1 E3 ubiquitin-protein ligase, and 1 unnamed protein (categorized into GO biological process, defense response to virus, DRV) in both guts and whole body. Six genes were confirmed to be significantly upregulated in guts samples (Fig. 5). Moreover, the fold changes obtained by the DEG were generally more extreme than those obtained by qRT-PCR. The inconsistency may be partially due to the lower sensitivity of qRT-PCR compared tosignificant DEG. In addition, the fold changes in guts were more significant than those in whole body. These results indicate that the transcriptome profiles of whitefly guts in response to TYLCV infection are a little different from the whitefly whole body. Nevertheless, qRT-PCR analyses confirmed the direction of change detected by DEG analysis, suggesting that DEG results are reliable.

## Discussion

### Altered receptors and transporters in whitefly gut after TYLCV infection

The GO and KEGG enrichment analysis showed that DEGs were significantly enriched in ‘Cargo receptor activity’ and “ATP-binding cassette (ABC) transporters”. Cargo receptor can selectively bind to an extracellular substance and deliver it into cell via endocytosis [21]. For example, cargo receptor ERGRC-53 is required for cellular glycoprotein trafficking of arenavirus, hantavirus, coronavirus, orthomyxovirus, and filovirus [22]. In human, the scavenger receptor class B type I and scavenger receptor B2 have been reported to be a receptor for the hepatitis C virus and enterovirus 71 separately [23, 24]. ABC transporters are members of a transport system superfamily and involved in diverse cellular processes such as maintenance of osmotic homeostasis, nutrient uptake, resistance to xenotoxins, antigen processing, bacterial immunity and pathogenesis [25]. Interestingly, one ABC superfamily transporter, multidrug resistance protein1 (MRP1), which is well-known to confer multidrug resistance to cancer cells through enhanced drug efflux [26], was also found in our DEGs. (c18467_g1: multidrug resistance-associated protein 1-like [*Diaphorina citri*], log_2_ ratio = 1.87) In addition, the downregulation of MRP2, which shows structural similarity to MRP1, suppresses the transport of various genotoxic substances in the liver during hepatitis virus infection [27]. As we know, virus receptors play essential roles in the early steps of viral infection. These regulated receptors and transporters might help TYLCV cross the whitefly epithelial cells.

### Perturbance of the cell cycle and DNA repair in response to TYLCV infection

Viruses depend on hosts machineries to replicate and express their genomes, and altering the host cell cycle is a common strategy of viruses inside the cell [28]. Perturbation of host cell cycle has been shown to be caused by geminivirus infection. For example, *Cabbage leaf curl virus* (CaLCuV) can activate genes expressed during S and G_2_ phases and inhibit genes active in G1 and M phases [29]. We noticed that one gene encoding cyclin D2 was upregulated in viruliferous guts. Cylin D2 (CCND2) is a member of the D-type cyclin family, which associates the extracellular signaling environment with cell-cycle progression [30]. Moreover, cyclin D2 was also upregulated in Hepatitis B virus-infected cells and has a positive role in HBV replication [31].

Virus infection can produce a large amount of exogenous DNA [32]. Several mammalian viruses have been shown to induce a cellular DNA damage response during replication, and in some cases, this response is required for optimal virus replication [33]. In addition, cell cycle checkpoints govern cell cycle progression in the presence of DNA damage and incomplete DNA replication [34]. The upregulated genes in cell cycle checkpoints suggest that TYLCV infection may induce DNA damage and affect cell cycle in whitefly gut cells. Interestingly, we also noted that all of the 10 upregulated genes encoding ubiquitin regulated E3 ubiquitin ligase activity. Furthermore, 5 of these genes accumulated in the G2 phase of the cell cycle. G2/M phase-specific E3 ubiquitin-protein ligase (G2E3) as a nucleo-cytoplasmic shuttling protein was implicated in the DNA damages response and cell cycle regulation [35], and then attenuated replicative stress [36]. Previous study also showed that *Beet severe curly top virus* (BSCYV) can induce RKP (a functional ubiquitin E3 ligase), which is able to interact with cell cycle inhibitor ICK/KRP proteins to regulate the cell cycle [37].

### Defense responses in whitefly gut in response to TYLCV

In our DEGs, 6 genes encoding AMPs were significantly upregulated. AMPs are evolutionarily ancient weapons that resist bacteria, fungi, viruses and any conceivable substance [38]. Previous study has shown that knottin genes are responsive to various stresses and related to TYLCV circulative transmission in the whitefly [10, 12]. Therefore, the observed upregulation of genes encoding the knottin proteins in our study are consistent with previous reports. For cellular responses, phagocytosis is a widely conserved cellular response and refers to the recognition, engulfment and intracellular destruction of invading pathogens and apoptotic cells [39]. Previous study in our laboratory has demonstrated that lysosome participate in autophagy, which could inhibit the efficiency of TYLCV transmission by whiteflies [11]. As stated above, all genes involved in humoral and cellular responses and most genes associated with lysosome were significantly upregulated, strongly suggesting that innate immune systems were activated in viruliferous whitefly guts.

For regulators of defense responses, the beta 1, 3-glucan recognition protein (βGRP), which can lead to melanotic encapsulation in insects [40], was upregulated in viruliferous gut. This result suggested that encapsulation might be activated after TYLCV infection. The altered genes encoding surface receptor were classified into three major classes; a) CD36 family (*n* = 4), b) epidermal growth factor (EGF)-like (*n* = 3), and c) Toll receptor (*n* = 2). CD36 family is class B scavenger receptor that participates in apoptotic cell removal in *Drosophila* embryos and in phagocytosis of *S. aureus* [41]*.* Interestingly, scavenger receptor has been noticed in GO enrichment analysis, but just 1 out of 4 of these CD36 genes was categorized into scavenger receptor activity. The EGF, a transmembrane protein, mediates phagocytosis, has been demonstrated with knockdown experiments, in a broad range of bacteria in *Drosophila* [42]*.* Spaetzle (markedly upregulated) is the ligand for Toll receptor, leading to Toll signally pathway controlling the potent resistances to bacterial, fungal, and viral infections [43]. Toll is one of the major signal transduction pathways (another one is Imd), used by antimicrobial responses in *Drosophila* [44].

The above results clearly show that following TYLCV infection, certain of immune responses of whitefly including humoral (antibacterial peptides) and cellular (phagocytosis, encapsulation) were activated. The regulators of phagocytosis (CD36, EGF) and encapsulation (βGRP, Toll receptor) were also activated. These immune responses may result in the degradation of virions, therefore, the activation of immune responses is probably the evolved strategy of the whitefly to protect itself from the deleterious effects of TYLCV infection.

### Intracellular signaling molecules and pathways involved in defense response

We also observed that a number of genes involved in signal transduction of the defense response were upregulated after TYLCV invasion. Ras is a key regulator of cell growth in all eukaryotic cells and positioned centrally in signal transduction pathways that respond to diverse extracellular stimuli [45]. The medfly homologues of Ras proteins have been shown to affect phagocytosis in response to lipopolysaccharide (LPS) and *E. coli* [46]*.* PI3K-Akt pathway is involved in apoptosis and autophagy in *Drosophila* [47, 48], as well as in medfly apoptosis [49]. Decreases in phagocytosis produced by pathway inhibitor indicated that PI3-kinase was required for internalization of bacteria [50]. Several studies have implicated involvement of MAPKs in insect innate immune responses [51, 52], and the activation of MAPK pathway contributes to efficient infection by baculoviruses [53]. Otherwise, several studies have documented that PKC plays an important role in regulating the receptor-mediated endocytosis of virus-receptors complexes [54–56], and specific inhibitor for PKC is highly effective in blocking *West Nile virus* (WNV) entry into mosquito cell line (C6/36) [57]. Here we found that all genes involved in the MAPK pathway and PKC were upregulated in DEGs.

The intracellular signaling molecules and pathways are organized as communication networks that process, encode and integrate internal and external signals. For example, activated Ras can directly interact with MAPK kinase kinase and activates distinct MAPK cascades (ERK, JNK, p38) [58]. Upon activation, the ERKs can stimulate the activity of Elk-1 transcription factor [59]. Blockage of Elk-1 like protein phosphorylation indicated that an Elk-1 like protein was candidate for the regulation of phagocytosis of bacteria [60]. Moreover, JNK was also reported to be involved in phagocytosis in mosquito cell line [61]. In our results, Ras, MAPKK7, JNK, Elk-1, even phagocytosis were upregulated. Evidently, this signaling network in whiteflies was activated by TYLCV.

## Conclusions

In summary, we present, for the first time, an analysis of the global transcription response of whitefly guts to TYLCV infection using high-throughput sequencing. Our data show that TYLCV can perturb the material transport and cell cycle of whitefly gut cells. This virus can also activate the humoral and cellular immune responses of whiteflies. Overall, our results reveal the complex interactions between begomovirus and whiteflies in the gut tissue. This study will provide a road map for future investigation of guts in plant virus-vector insect interactions and may offer hints for the discovery of novel and specific molecular targets for the control of whitefly transmitted virus.

## Methods

### Whitefly cultures and virus clone

The culture of *B. tabaci* MEAM1 (mtCO1 GenBank accession no. GQ332577) was maintained on cotton (*Cossypium hirsutum* cv. Zhe-Mian 1793), which is a non-host plant of TYLCV. Whiteflies were reared in a climate chamber at 26 ± 1 °C, LD 14:10 h and 60 ± 10% relative humidity. To obtain virus-infected plants, clones of TYLCV isolate SH2 (GenBank accession no. AM282874.1) were inoculated into tomato (*Solanum lycopersicom* L.cv. Hezuo903) at 3–4 true-leaf stage as previously described [62], TYLCV-infected and uninfected tomato plants were cultivated to 6–7 true-leaf stage when used in experiments. All plants were grown in a greenhouse at 20–30 °C, LD14:10 h and 50–70% relative humidity.

### Whitefly treatments and sample collection

To prepare viruliferous and nonviruliferous whiteflies, approximately 2000 newly emerged (0–48 h) adult whiteflies were separately collected and released onto healthy tomato plants in different cages for 48 h. Then the whiteflies were transferred onto virus-infected and uninfected tomato plants for 48 h, respectively. After that, they were separately transferred onto another tomato (*Solanum lycopersicom* L.cv. Zheza502), which is a resistant variety to TYLCV [11], to eliminate effects of host differences on whiteflies. After 24 h, the whiteflies were used for gut dissection. For sample collection, approximately 1000 guts were dissected from viruliferous and nonviruliferous whiteflies in PBS respectively, and frozen in liquid nitrogen, stored at − 80 °C until RNA isolation. Additionally, all the guts were dissected in 36 h to minimize the impact of time on the results.

### RNA isolation and cDNA library preparation

Total RNA was extracted from pools of approximately 1000 guts using the Absolutely RNA Nanoprep kit (Agilent, USA) according to the manufacturer’s manual with slight modifications [63]. RNA quantity and quality were assessed using 1% agarose gels, Qubit® 3.0 Flurometer (Life Technologies, CA, USA) and the RNA Nano 6000 Assay Kit of the Agilent Bioanalyzer 2100 system (Agilent Technologies, CA, USA). RNA (700 ng per sample) was used to generate adaptor-ligated double- stranded cDNA libraries for RNA-Seq using the NEBNext® UltraTM RNA Library Prep Kit for Illumina® (New England Biolabs, USA) following the manufacturer’s protocol. The fragment size and concentration of resultant libraries were carried out using the Agilent Bioanalyzer 2100 system (Agilent Technologies, CA, USA) and StepOnePlus™ Real-Time PCR System (Library valid concentration > 10 nM).

### Transcriptome sequencing and assembly

The cDNA libraries were sequenced for 150 bp paired-end reads using Illumina Hiseq 4000 platform at Annoroad Gene Technology Company (Beijing, China). The total sequencing amount was 8 G for each library. To ensure the quality of data used in further-analysis, raw data were transformed into clean data through removing adaptor-polluted reads (more than 5 adaptor-polluted bases), low-quality reads (more than 15% bases with Phred threshold score ≤ 19), and reads with unknown sequences ‘N’ accounting for more than 5% using Perl scripts. As for paired-end sequencing data, both reads were filtered out if any read of the paired-end reads are adaptor-polluted. The assembler Trinity was used for de novo assembly [64]. We quantified the proportion of the clean reads that mapped to each assembly using Bowtie (v2.2.3). To test the completeness of the assemblies, we mapped the presence of highly conserved genes with 2748 core proteins from conserved regions of eukaryotes. TransDecoder was used to identify the candidate coding regions within transcript sequences, and Trinotate was used for performing the functional annotation of unigenes and ORFs.

### Analysis of differential gene expression

The clean reads from viruliferous and nonviruliferous guts were separately mapped back to the assembled unigenes. For gene expression analysis, reads per kilobase million Mapped Reads (RPKM) was calculated to estimate the expression level of genes in each sample. RPKM can eliminate the effect of sequencing depth and gene length on gene expression levels, which facilitates the comparison of the number of transcript levels generated between samples. DEGseq (v1.18.0) was used to identify differentially expressed genes (DEGs) between the viruliferous and nonviruliferous guts. Genes with q ≤ 0.05 (adjusted *p*-value) and log_2_ ratio ≥ 1 were considered differentially expressed.

### GO and KEGG pathway analysis

To obtain an overview and further understand the biological functions of genes, all DEGs were subjected to GO functional annotation using Blast2GO and mapped to terms in KEGG pathway database using KOBAS. Enrichment analysis was used to identify the GO terms and significantly regulated KEGG pathways. We selected a corrected q ≤ 0.05 as the threshold to determine significant enrichment of the gene sets.

### qRT-PCR analysis

To confirm the result of the DEG analyses, we measured the expression of 10 selected genes using comparative C_T_ (ΔΔC_T_) Real-time Quantitative PCR with β-actin as the internal control gene. We prepared viruliferous and nonviruliferous whiteflies as described above and gut total RNA was extracted from pools of approximately 70 guts. Three biological replicates were conducted at the same time. Moreover, 25 whiteflies were collected (24 h after beginning of the gut dissection) from each replication to compare the differences in gene expression between the gut and the whole body. Total RNA was extracted using the TRI-reagent method (Life Technologies, USA). RNA was reverse transcribed using the SYBR® PrimeScript™ RT-PCR Kit II (Takara, Japan). qRT-PCR was performed using PTC-200 Thermocycler (Bio-Rad, USA) with SYBR-Green detection (SYBR® Premix Ex Taq™ II, Takara, Japan). The primer sequences are provided in Additional file 2: Table S2.

## Additional files

Additional file 1: Table S1. DEGs involved in Lysosome. Displayed are the fold changes of DEGs involved in Lysosome of viruliferous guts in comparison to nonviruliferous guts. ^a^The function of the homologous gene. ^b^FC, fold change (log2 ratio) of gene expression. (XLSX 8 kb)

Additional file 2: Table S2. Sequences of primers used in the study. Displayed are the sequence of the primers used for qRT-PCR. Primers were synthesized by GenScript (Nanjing, China). (XLSX 9 kb)
